# Salinity drives the biogeography and functional profiles of the oyster-associated microbiome along the Chinese coastline

**DOI:** 10.1093/ismeco/ycaf080

**Published:** 2025-05-09

**Authors:** Liusheng Lei, Xin Li, Linhao Chen, Xiaolong Wang, Qingbin Yuan, Zeyou Chen, Daqing Mao, Yi Luo, Huai Lin

**Affiliations:** State Key Laboratory of Water Pollution Control and Green Resource Recycling, School of the Environment, Nanjing University, Nanjing 210093, Jiangsu Province, China; State Key Laboratory of Water Pollution Control and Green Resource Recycling, School of the Environment, Nanjing University, Nanjing 210093, Jiangsu Province, China; State Key Laboratory of Water Pollution Control and Green Resource Recycling, School of the Environment, Nanjing University, Nanjing 210093, Jiangsu Province, China; State Key Laboratory of Water Pollution Control and Green Resource Recycling, School of the Environment, Nanjing University, Nanjing 210093, Jiangsu Province, China; State Key Laboratory of Water Pollution Control and Green Resource Recycling, School of the Environment, Nanjing University, Nanjing 210093, Jiangsu Province, China; Ministry of Education Key Laboratory of Pollution Processes and Environmental Criteria, College of Environmental Science and Engineering, Nankai University, Tianjin 300350, Tianjin Province, China; School of Medicine, Nankai University, Tianjin 300310, Tianjin Province, China; State Key Laboratory of Water Pollution Control and Green Resource Recycling, School of the Environment, Nanjing University, Nanjing 210093, Jiangsu Province, China; State Key Laboratory of Water Pollution Control and Green Resource Recycling, School of the Environment, Nanjing University, Nanjing 210093, Jiangsu Province, China

**Keywords:** carbon, nitrogen and sulfur cycles, Chinese coastline, functional taxa, oyster, salinity

## Abstract

Understanding the influence of environmental factors on the taxonomic and functional profiles of microbial communities is critical for assessing ecological health. In this study, we perform a large-scale field survey and microcosm experiment to investigate the effects of environmental heterogeneity on the microbial communities and functional profiles of oysters along the Chinese coastline. We found that salinity altered the spatial distribution of oyster-associated microorganisms and their functional profiles between the southern and northern regions. Specifically, the northern regions, with optimal salinity (18.3 part per thousand), exhibited a higher abundance of dominant functional microorganisms, more stable microbial networks, and enhanced carbon, nitrogen, and sulfur biogeochemical cycles than the southern regions. Moreover, metabolic mutualism among key taxa, such as *Vibrio*, *Pseudomonas*, and *Shewanella*, was identified as crucial for the coupled carbon, nitrogen, and sulfur cycles. These results suggest that salinity-driven microbial interactions and compositions play predominant roles in structuring the spatial heterogeneity of the functional profiles of oyster-associated microorganisms. Microcosm experiments further confirmed that moderate salinity, a crucial indicator of climate change, regulates and enriches the primary functional profiles of oyster-associated microorganisms. Overall, this study highlights how environmental conditions shape oyster-associated microbial and functional traits along the Chinese coastline, raising concerns about the impact of anthropogenic activities, such as climate change, on marine ecological functions.

## Introduction

Coastal marine ecosystems are habitats for highly diverse communities that contribute to up to 77% of global ecosystem service [1]. They provide a range of ecologically and economically critical ecosystem services, including nutrient cycling, coastal protection, and fisheries enhancement [2]. Notably, coastal marine ecosystems are critical zones for material exchange and energy transformation. For instance, coastal marine ecosystems act as primary carbon (C) reservoirs, actively participating in the fixation and release of C, thereby influencing the C cycle in global ecosystems [3]. Hence, safeguarding coastal marine ecosystems is of paramount importance for facilitating material circulation and energy flow on Earth, especially the C, nitrogen (N), and sulfur (S) cycles, consequently contributing to global ecosystem health.

Oysters as the typical bivalves, represent the largest group of described coastal marine organisms and play a vital role in the functioning of coastal marine ecosystems. As part of the coastal marine ecosystem, these marine animals are widely distributed along the 18 000-km coastline of China, primarily from north to south [4]. More important, oysters ingest up to 5 L of seawater per hour through their gills [5, 6]. During filter feeding, suspended microorganisms in the seawater enter and colonize their bodies [5, 6]. Hence, oysters are teeming with microorganisms that inhabit the hosts’ blood, mantle, gills, and gut [1, 7]. The host-associated microbiome has recently gained recognition as a major contributor to ecosystem services and host functionality [8, 9]. Notably, these microorganisms participate in biogeochemical cycling through the remineralization of organic material and the transformation of C, N, and S between inorganic forms. For instance, they are involved in the N cycle, comprising nitrification, denitrification, and both aerobic and anaerobic ammonia oxidation [9, 10]. They can also convert hydrogen sulfide into oxidized or partially oxidized forms, such as sulfate and thiosulfate. Given their substantial potential, we believe that the oyster-associated microorganisms play an important role in coastal marine ecosystem functioning which have largely been overlooked. Furthermore, China is the leading oyster aquaculture country worldwide, and the spatial distribution of oyster-associated microorganisms and their functional profiles along China’s coastline, especially in terms of C, N, and S cycles, remain largely unexplored.

Uncovering the factors that affect the taxonomic and functional profiles of host-associated microbiome is essential for exploring biogeography and understanding and predicting how organisms respond to changing environments [11, 12]. A growing body of research has highlighted the importance of environmental factors in shaping microbial communities and influencing their ecological functions [13–18]. For example, temperature plays a crucial role in structuring soil-borne bacterial communities and altering the associations between protists, bacteria, and viruses [14]. With the increasing salinity stress induced by the freshwater-to-seawater transition, the microbial structure and microbial ecological networks change significantly [13]. In particular, the microbiota of coastal marine organisms is highly susceptible to external environmental disturbances [19–21]. These microbial changes may alter their composition, disrupt functional processes, and ultimately affect ecosystem functioning [22, 23]. However, few studies have investigated the impact of environmental factors on the functioning of host-associated microbiome [24] which play an important role in coastal ecosystem processes. Consequently, the mechanisms underlying the effects of environmental heterogeneity on the spatial distribution of the taxonomic and functional profiles of oyster-associated microorganisms remain largely overlooked.

Given the hypothesis that environmental heterogeneity determines biogeography and subsequently influences the functional profiles of oyster-associated microbiome, we conducted a cross-shoreline field survey and laboratory microcosm experiments. We characterized oyster-associated microbial diversity, composition, network, and functional profiles, including C, N, and S cycles, along Chinese coastline. We further elucidated the mechanisms underlying the spatial heterogeneity of oyster-associated microbiome and their functional profiles. Finally, empirical evidence for the salinity-modulated spatial distribution of the taxonomic and functional profiles of oyster-associated microbiome was obtained through microcosm experiments that artificially manipulated salinity gradients.

## Material and methods

### Study sites, sample collection and in-*situ* measurement of environmental variables

**Figure 1:**
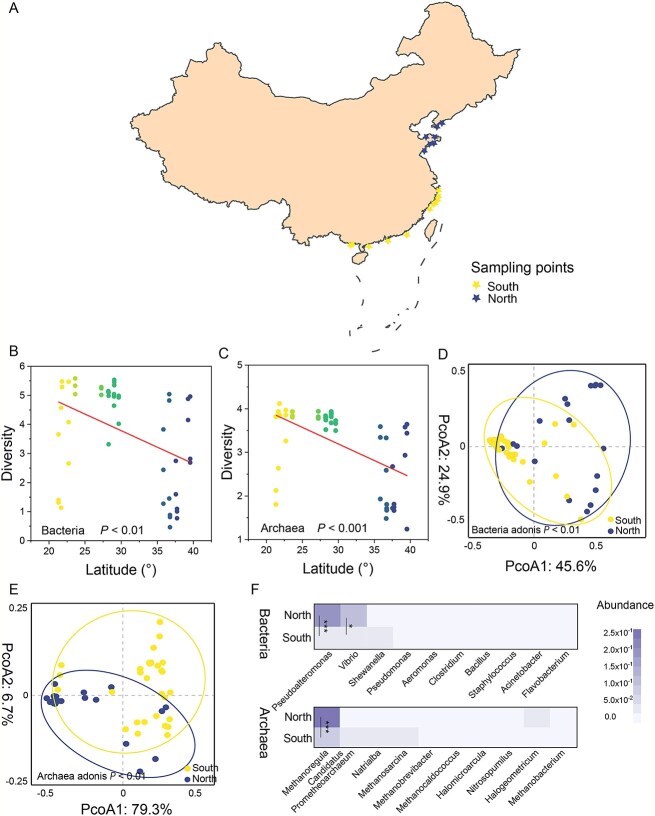
The diversity, structure, and composition of oyster-associated microorganisms along Chinese coastline. (A) Geographic distribution of sampled sites. The sites from which the oyster samples were collected is highlighted with asterisk. All sites are marked and divided into southern (latitude *<*32°N) and northern (latitude *>*32°N) regions. This separation corresponds to the Qinling Mountains-Huaihe River line (latitude ≈ 32°N), an important geographical boundary in Chinese north–south regions. (B and C) spatial distribution of bacterial and archaeal diversity along the latitude of sampling locations. (D and E) PCoA plots based on Bray–Curtis distance show the patterns of bacterial and archaeal structure between northern and southern regions. (F) Bacterial and archaeal composition at genus level. Significant differences: ^*^P < 0.05 and ^***^P < 0.001.

In accordance with three key criteria: (i) representativeness, ensuring that the selected locations reflect the major, high-production mariculture regions in China; (ii) sample identity, guaranteeing that oysters of similar age and variety were collected to minimize variability; and (iii) sample accessibility, ensuring that samples could be easily obtained and preserved for microbial analysis, we collected oysters from 18 representative oyster mariculture sites along the 18 000-km coastline of China (Fig. 1A). These sampling sites represented the four main oyster farming regions in China, which also have substantial oyster production cover major oyster farming province including Liaoning (S1-S2), Shandong (S3-S7), Zhejiang (S8-S13), Fujian (S14), Guangdong (S15-S16), and Guangxi (S17-S18) (Table S2), accounting for ˃90% of total production of oysters in China [25]. According to the Qinling Mountains–Huaihe River Line, an important geographical boundary in Chinese north–south regions, we divided the sample sites into northern (S1–S7) and southern (S8–S18) regions. The oyster species included *Crassostrea gigas*, *Crassostrea angulate*, *Crassostrea hongkongensis*, and *Crassostrea sikamea*. Three adult oysters were randomly collected from each site when they reached a shell height of 6–12 cm in and 7–12 months old (Table S2) [26]. After collection, the all 54 oysters were promptly kept in an icebox, and transported to the lab. After field sampling, oysters were ground using tissue grinder in preparation for microbial DNA extraction.

Seawater (5 L) was also collected from each sampling site. We measured seawater temperature, pH, salinity, Fe^3+^, and NH_4_^+^-N using DZ-C aquaculture water quality detector (Qingdao Juchuang Environmental Protection Group Co., LTD). Moreover, using LZ-W200 portable touch multi-parameter water quality detector (Nanjing Quantum Chemical Technology Co., LTD), we determined chemical oxygen demand (COD), total nitrogen (TN), and total phosphorus (TP) in-*situ*.

### DNA extraction and sequencing

All oyster were washed in shells with 75% ethanol and carefully opened, leaving animal intact. The whole tissues samples, without distinguishing between gut or intracavitary contents, were homogenized. Total microbial DNA was extracted from treated oysters using E.Z.N.A. Stool DNA Kit (Omega Biotek, CA), following the manufacturer’s instructions. DNA concentration and purity were measured with Nanodrop 2000 (Thermo Scientific, Waltham, MA). Metagenomic libraries were generated using VAHTS® Universal Plus DNA Library Pren Kit for Illumina and were subjected to 150 bp paired-end sequencing through Illumina Nova-seq 6000 platform.

### Bioinformatic analysis

Trimmomatic (v0.33) with the default parameter [27] was conducted to quality-control of raw data. The filter data was mapped to oyster genome (GCA_000297895) using bowtie2 to receive clean reads. Kraken2 (v2.1.3) and Bracken (v1.0.0) with the default parameter [28] was used to annotate the bacterial and archaeal communities and calculate their abundance, respectively.

Each of oyster samples was independently assembled by MEGHIT (v1.29) using default parameters. Genes of all obtained contigs were predicated using Prodigal. A non-redundant gene catalog for all samples was constructed using CD-HIT; from this set, genes with >90% overlap and >95% nucleic acid similarity were removed as redundancies. Functional assignment of the non-redundant set of genes was annotated by Diamond on National Center for Kyoto Encyclopedia of Genes and Genomes (KEGG) (https://www.genome.jp/kegg/) and Non-supervised Orthologous Groups (eggNOG) (http://eggnog5.embl.de) databases. The abundance of each functional gene was calculated based on the counts per million (CPM).

We further constructed the oyster-associated metagenome-assembled genomes (MAGs) using MetaWRAP (v1.3.2) with default parameters [29]. The quality of each MAGs was assessed using CheckM (v1.2.1) [30]. The good-quality MAGs (completeness >50% and contamination <10%) were kept for constructing the non-redundant MAGs set using dRep (v3.4.0) [31]. CoverM (v 0.7.0) (https://github.com/wwood/CoverM) and GTDB-Tk (v2.1.1) [32] were used for abundance and taxonomic classifications of this catalog, respectively. The functional gene annotation for this set were conducted using KEGG and eggNOG databases.

### In vitro microcosm experiment

The microcosm experiment was performed in oysters independent of the large-scale survey presented above to assess the effects of different salinity conditions on the taxonomic and functional profiles of oyster-associated microorganisms and enable us to explore the underlying mechanisms of salinity induces the spatial pattern of functional profiles of oyster-associated microorganisms independently of the data used. The oysters (*Crassostrea angulata*; length: 7.6 ± 1.0 cm, width: 4.2 ± 0.4 cm, height: 2.8 ± 0.6 cm) for microcosm construction were collected from mariculture farms and promptly transported to experimental tanks for acclimatization (2 weeks). Prior to transfer, all oysters are carefully inspected and cleaned to remove parasites.

Three salinity gradients (15, 25, and 30 part per thousand, ppt) were set up in this microcosm experiment according to the measured salinity in this study and the reported the high salinity (29–33 ppt) [33]. After acclimation, these oysters were cultured in 20-L tanks per treatment (15 oysters per tank) over a 10-day exposure period. Oysters fed microalgae (*Chlorella pyrenoidosa*) at a daily ratio equal to 7%–8% dry-weight-algae/dry-weight-oyster [34]. The tank seawater was natural seawater filtered with 0.22 um filter membrane and well mixed via an aeration system. Seawater, food, and wastes were renewed daily. The cultured temperature was 17 ± 1°C and under a 12/12 h light/dark cycle [35]. Every day, salinity was measured daily and dead oysters were removed. After incubation of 10 day, five oysters were randomly collected from each tank.

We extracted microbial DNA from the oysters and performed amplicon sequencing at Biomarker Technologies Co., LTD (Beijing, China) on the Illumina NorvaSeq 6000 platform with 150 bp paired-end technology to assess the variation in bacterial communities along the salinity gradients. To assess the response of functional profiles of oyster-associated microorganisms to the salinity gradients, we identified the key genes related to C (*naglu*, *manB*, *abfA*, *amyA*, *accA*, *aclB*, *frdA*, and *korA*), N (*glnA*, *napA*, *narG*, *nirK*), and S (*dsrAB* and *soxY*) cycles using qPCR (Table S1) [36, 37]. The detailed information was shown in supplementary information Sections I and II. We also explore the carbon metabolism function of microbial communities using Biolog™ Ecoplates technology [38] and the detailed information was provided in supplementary information Section III.

### *Vibrio* strain isolation and growth curve measurement

*Vibrio* strains were high abundant, well associated with other genera, and exhibit abundant functional potential in this study (Figs. 1F, 5B, 4, and S4), we thus focused on *Vibrio* to evaluate the effect of salinity on them and better understand their roles within oyster-associated microbial communities. All collected oysters were ground, serially diluted with sterile phosphate-buffered saline, and *Vibrio* strains were isolated using thiosulfate citrate bile salts sucrose agar medium. The growth curves of *Vibrio* strains were performed under a range of salinity categories including 0, 5, 10, 15, 20, 25, 30, 35, 40, 45, 50 ppt. OD_600_ was monitored at each 30 min. Each condition was repeated six times. Growth curve parameters were calculated using R package “Growthcurve”. The detailed information was provided in Sections IV and V.

### Statistical analysis

The Shannon index, principal coordinate analysis (PcoA) and analysis of similarity were conducted using “vegan” package in R. Network analysis was performed using Gephi v0.98 based on Pearson’s correlation coefficients (R > |0.9|, *P* < 0.01) to elucidate the interactions among microbial communities at the genus level. The phylogenetic tree of MAGs was constructed using IQTree [39]. Mantel test was used to determinate the factors affecting the microbial communities and their functional potential. Student’s *t*-test was conducted to assess significant differences using SPSS 25. The schematic diagrams were drawn using BioRender (https://app.biorender.com) with full publishing rights.

## Results

### Salinity-driven spatial patterns of oyster-associated microbial communities

**Figure 2:**
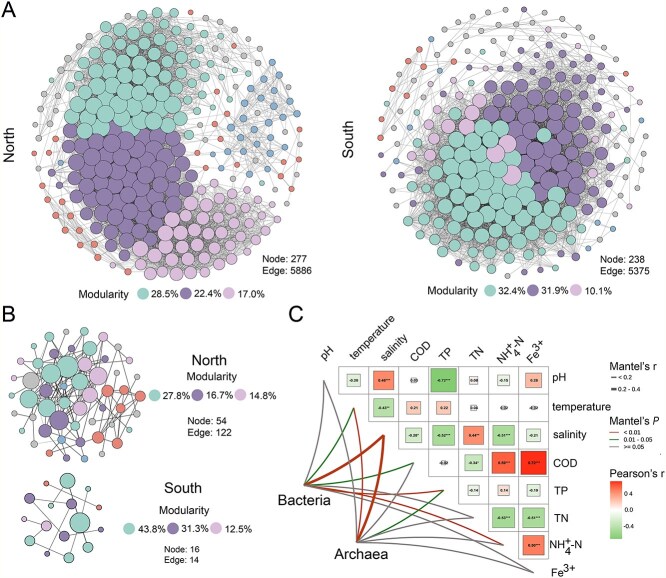
Salinity alters the co-occurrence networks of oyster-associated microorganisms between southern and northern regions. (A and B) The co-occurrence networks of bacterial and archaeal communities according to Pearson’s analysis. The nodes are colored according to modularity and node size is proportional to node degree. A connection served as a significant correlation (R > |0.9|, *P* < 0.01). (C) The correlation analysis between environmental factors, mainly including salinity, temperature, pH, and TN, and composition of microbial community based on the mantel tests. Color gradient and circle size denote Pearson’s correlation coefficients. The color of the line represents the significance of the differences (*P*-values). The size of the line represents correlation coefficients (mantel’s r). Asterisks in the circle denote for different significance levels at ^*^P < 0.05, ^**^P < 0.01, and ^***^P < 0.001.

Oyster-associated microbial communities are spatially distributed. Using metagenomic sequencing, we explored the distribution of oyster-associated microbiome collected from 18 representative oyster mariculture sites along the Chinese coastline (Fig. 1A). Based on taxonomic analysis, we found that bacterial and archaeal richness gradually declined with latitude from south to north (Fig. 1B and C), suggesting the biogeography of oyster-associated microbiome along the Chinese coastline. Moreover, a clear clustering of the bacterial and archaeal community was observed, with distinct variations in the oyster-associated microbial composition between the northern and southern regions (Fig. 1D and E; adonis *P* < 0.01). This pattern coincided with the Qinling Mountains–Huaihe River geographical line (latitude ≈ 32^°^N), a key geographical boundary separating northern and southern Chinese regions. Therefore, this natural boundary division was considered for further analyses. Our results revealed a notable shift in microbial profiles from northern to southern regions, including alterations in diversity, composition, and network interactions. Dominant bacteria, including *Pseudoalteromonas* (0.04%–94.1%) and *Vibrio* (0.94%–85.6%), as well as archaea, such as *Methanoregula* (0.82%–76.0%), were significantly reduced by 80.3%, 64.7%, and 67.0%, respectively, along the transition from northern to southern latitudes (Figs. 1F and S1; *P* < 0.05). Consistent with the differences in microbial composition, the microbial networks exhibited distinctions. The southern regions showed a decrease in the number of bacterial (north: south = 277: 238) and archaeal nodes (north: south = 54: 16) compared to those of the northern regions (Fig. 2A and B). This indicates a significant variation in the co-occurrence patterns of oyster-associated microorganisms between the northern and southern regions. Compared to the northern regions, the southern regions showed a reduction in the number of bacterial (north: south = 5886: 5375) and archaeal (north: south = 122: 14) edges, suggesting that the southern regions harbored a more unstable microbial network. Moreover, the southern regions exhibited a more centralized modular structure in both bacterial (top three modules, south: north = 74.4%: 67.9%) and archaeal networks (top three modules, south: north = 87.6%: 59.3%), suggesting potential changes in microbial ecological functions compared to those of the northern regions. The spatial heterogeneity of physicochemical properties may result in variations in the spatial distribution of oyster-associated microbiome. We measured a range of physicochemical properties in oyster mariculture areas, such as salinity, temperature, and pH, and observed significant differences in salinity between the northern (18.3 ppt) and southern (13.3 ppt) regions (Fig. S2 and Table S4; *P* < 0.05). Interestingly, based on the Mantel test, we found that salinity was markedly correlated with oyster-associated microorganisms, including bacteria and archaea (Fig. 2C; *P* = 0.01). Meanwhile, we also found that salinity was significantly correlated with bacterial and archaeal networks (Fig. S12; *P* < 0.05). Therefore, we speculate that changes in salinity offer explanatory insights into the observed variances in the spatial distribution of oyster-associated microbial taxa.

### Salinity influences the spatial distribution of oyster-associated microbial functional profiles

Salinity caused the southern regions to carry more unstable and centralized microbial networks and a low abundance of dominant genera, which could indicate that the functional processes of oyster-associated microorganisms in these regions were adversely affected. To further explore the spatial distribution of the functional profiles of oyster-associated microorganisms, we first identified their functional traits along the Chinese coastline. In this study, 22 functional pathways were identified in all samples. These pathways were predominantly associated with metabolism (Fig. S3). Among these, the most abundant metabolic pathways were carbohydrate metabolism (11.9%–15.0%), amino acid metabolism (8.6%–11.7%), nucleotide metabolism (3.7%–7.1%), metabolism of cofactors and vitamins (3.6%–4.8%), and energy metabolism (3.0%–5.8%). In particular, along the transition from the northern to southern regions, the abundance of metabolic processes, such as energy metabolism, exhibited a decreasing trend (Fig. S3). This trend was consistent with variations in microbial composition and networks (Figs. 1F, 2A and B), which may influence the biogeochemical cycles of C, N, and S.

**Figure 3:**
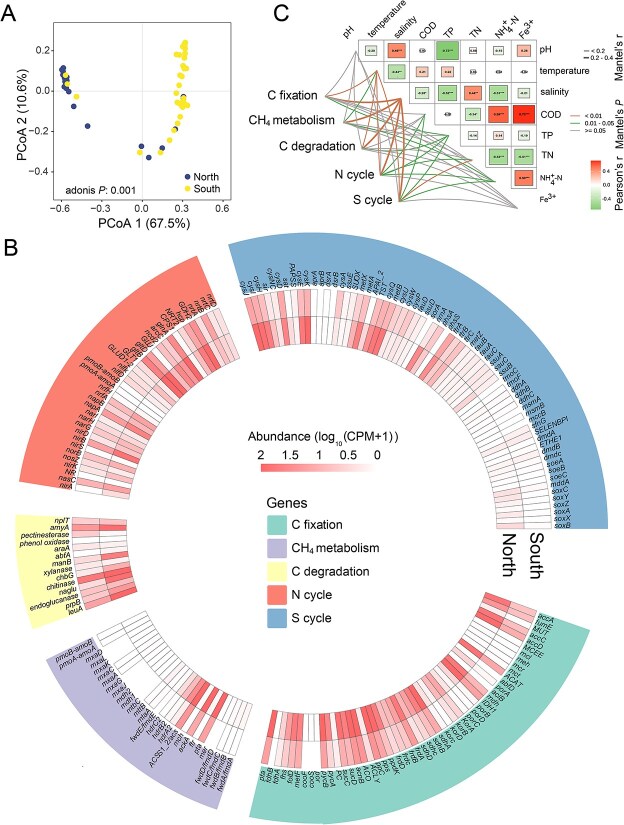
Salinity influences the functional profiles of oyster-associated microorganisms along Chinese coastline. (A) PCoA plot based on Bray–Curtis distance show the patterns of functional profiles between northern and southern regions. (B) Heatmap of these functional genes comprising C, N, and S cycles, in southern and northern mariculture regions. Genes belonging to the same function are depicted using the consistent color. C cycle includes C fixation, C degradation, and CH_4_ metabolism. C: Carbon; CH_4_: Methane; N: Nitrogen; S: Sulfur. (C) The correlation analysis between environmental factors, mainly comprising salinity, temperature, pH, and TN, and functional profiles based on the mantel tests. Color gradient and circle size denote Pearson’s correlation coefficients. The color of the line represents the significance of the differences (*P*-values). The size of the line represents correlation coefficients (mantel’s r). Asterisks in the circle denote for different significance levels at ^*^P < 0.05, ^**^P < 0.01, and ^***^P < 0.001.

Functional genes related to C (C fixation, C degradation, and CH_4_ metabolism), N, and S cycles exhibited spatial differences from southern to northern transition. The functional gene profiles of the oyster-associated microbiome were clearly separated between northern and southern regions (Fig. 3A; adonis *P* = 0.001). Moreover, we observed that the fixation (e.g. *acnB*, *ppC*, and *ACO*) and degradation (e.g. *chitinase*, *leuA*, and *amyA*) of C were active due to the abundance of relevant genes (Fig. 3B), implying that oyster-associated microorganisms played an important role in the C cycle. Compared to the northern regions, the southern regions exhibited a significant reduction in the abundance of these relevant genes, which was consistent with the lower abundance of dominant genera and more unstable, centralized microbial networks (Figs. 1F, 2A and B). These results suggest that the southern regions potentially influence C fixation and degradation, thereby further harming the C budget and cycle. Moreover, the southern regions exhibited a reduced abundance of key genes related to the C (e.g. *IDHl*, *sdhA*, *ppC*, *pycB*, *pta*, *ACSS/acs*, and *hdrA2*), N (e.g. *nirB*, *napA*, *gltBD*, *glnA*, *ncP*, and *GPH2*), and S (e.g. *cysIJHEKQ and metA*) cycles, which were predominantly responsible for the C, N, and S processes. These findings suggest that the southern regions may suppress the biogeochemical processes of oyster-associated microbiome, compared to those in the northern regions (Fig. 3B), thereby jeopardizing coastal marine ecosystem health. Overall, these results highlight the spatial heterogeneity of the functional profiles of oyster-associated microbiome. The spatial heterogeneity of physicochemical properties may result in variations in the spatial distribution of functional profiles of oyster-associated microbial communities. We observed significant correlations between salinity and the biogeochemical processes of oyster-associated microbiome, including the C (C fixation, C degradation, and CH_4_ metabolism), N, and S cycles, according to the results of the Mantel test (Fig. 3C; Mantel’s *P* < 0.01). This indicated that salinity was the primary factor influencing these functional processes. Meanwhile, we observed significant differences in salinity between the northern (18.3 ppt) and southern (13.3 ppt) regions (Fig. S2; *P* < 0.05). Collectively, these results suggest that salinity regulates the spatial distribution patterns of the functional profiles of oyster-associated microbial communities.

### Salinity-driven spatial variation in functional profiles: Altering associated functional microorganisms

**Figure 4:**
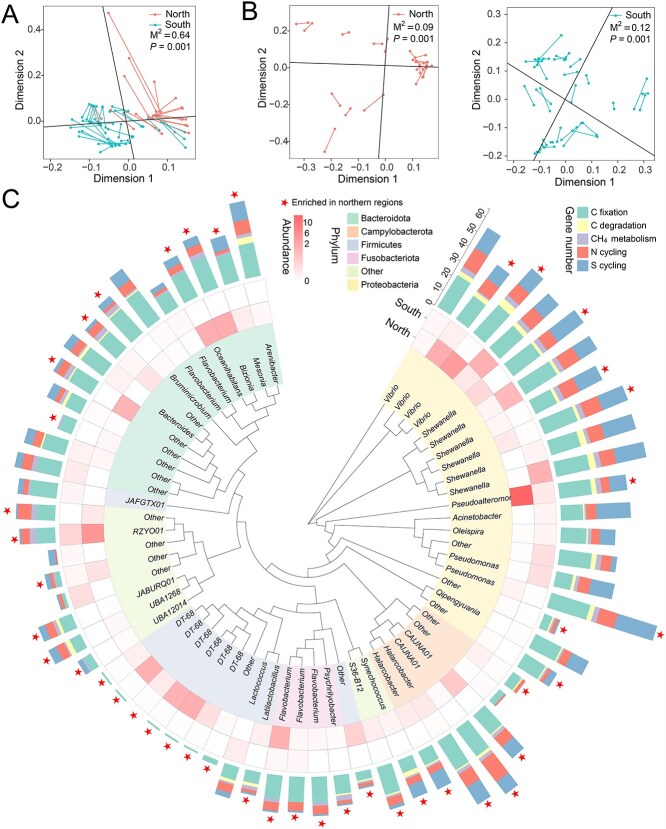
Correlation of microbial communities with functional profiles. (A) Procrustes analysis of functional genes and bacterial communities at the scale of the entire coastline. (B) Procrustes analysis of functional genes and bacterial communities at northern and southern regions. (C) Abundance of good-quality metagenomic assembled genomes (MAGs) and their carried the carbon, nitrogen, and sulfur cycling genes. The phylogenic tree of the MAGs in all samples. Among these genomes, genes related to C fixation, C degradation, CH_4_ metabolism, N cycle, and S cycle are identified. The taxa belonging to the same phylum represented by the identical color in the leaves of the phylogenetic tree. C: Carbon; CH_4_: Methane; N: Nitrogen; S: Sulfur.

To illustrate the underlying mechanisms by which salinity induces the spatial pattern of the functional profiles of oyster-associated microorganisms, we explored the associations between the taxonomic and functional profiles of oyster-associated microorganisms using Procrustes analysis. At the scale of the entire coastline, Procrustes analysis showed a significant correlation between the functional profiles and microbial composition (Fig. 4A; M^2^ = 0.64, *P* = 0.001). Procrustes analysis further proved that the functional profiles were affected by microbial composition in both the northern (M^2^ = 0.09, *P* = 0.001) and southern (M^2^ = 0.12, *P* = 0.001) regions (Fig. 4B). These results indicate that regional microbial variations may lead to spatial differences in functional traits. Combined with the microbial composition (Fig. 1F) and microbial networks (Fig. 2A and B), salinity leads to spatial differences in functional traits.

To further validate the contribution of microbial composition, especially functional microorganisms, to the spatial distribution of these functional profiles during the transition from the northern to southern regions, we reconstructed metagenome-assembled genomes (MAGs) from all samples. In this study, we identified 63 MAGs that contain at least one functional gene, including those involved in C, N, and S cycling genes (Figs. 4C and S4). Among these MAGs, *Vibrio* strains encoded abundant genes related to the fixation and degradation of C, such as the reductive citrate cycle (e.g. *sdhABCD* and *sucCD*), starch degradation (*amyA*), and chitin degradation (*chitinase*). *Vibrio* strains also encoded genes related to the N cycle, such as N fixation (e.g. *nifK*) and N reduction (e.g. *napAB* and *nirBD*), which are essential for the C and N cycles in oyster-associated microorganisms. *Shewanella* strains identified a series of C (e.g. *frdABC*, *leuA*, and *prpB*), N (e.g. *napAB* and *glnA*), and S (e.g. *cysADEHIJKNP* and *metAB*) cycling genes. In addition, *Pseudomonas* strains carried a range of functional genes related to C (e.g. *accABCD*, *leuA*, and *prpB*), N (e.g. *nasC*, *nirBD*, and *glnA*), and S (e.g. *cysADEHIJKNP* and *metBXZ*) cycles. These results suggest that metabolic mutualism among these functional MAGs plays a critical role in coupled C, N, and S biogeochemical cycles. Furthermore, according to Mantel tests, salinity exhibited the most significant correlation with functional microorganisms (Fig. S5). This indicates that salinity influenced the spatial distribution of functional taxa, which is consistent with our analysis of microbial composition (Fig. 2C). Specifically, most MAGs (43/63) were more abundant in the northern regions than in the southern regions, and their reconstructed metabolic gene related to C (53/58), N (23/29), S (26/45) cycles also exhibited enhanced representation (Fig. S6), which may have contributed to the enrichment of functional profiles in the northern regions. In summary, these results suggest that salinity affects the distribution of functional microorganisms, such as *Vibrio*, *Shewanella*, and *Pseudomonas*, as well as their metabolic pathways, driving spatial heterogeneity in the functional traits of oyster-associated microorganisms.

### Optimal salinity shapes the spatial heterogeneity of oyster-associated microbiome functional profiles

**Figure 5:**
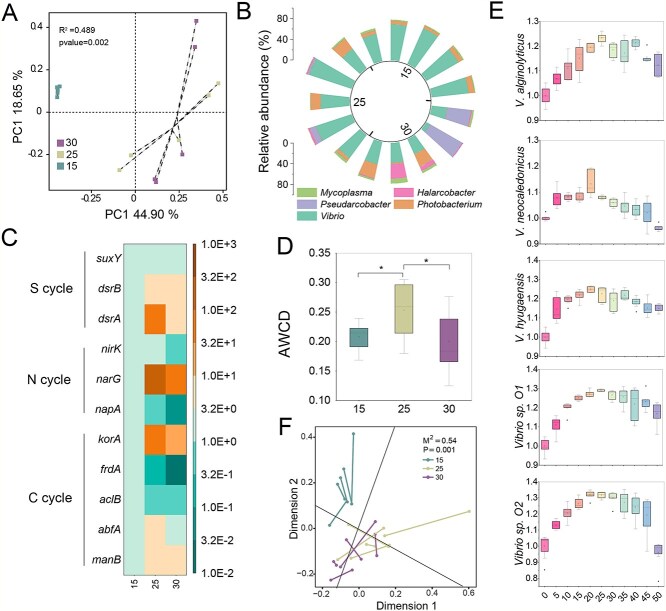
The effects of salinity on the taxonomic and functional profiles of oyster-associated microbial communities in vitro microcosm experiments. (A) Bacterial structure at OTU level. (B) Bacterial composition at genus level. (C) The fold change of functional genes related to C, N, and S cycling genes. (D) The average well-color developments (AWCD) of carbon sources using the biolog™ Ecoplates. Significant difference: ^*^P < 0.05. (E) The fitness of *vibrio* strains at the different salinity level (0–50 ppt). The fitness of strains is calculated based on the area under the curve. (F) Procrustes analysis of functional genes and bacterial communities.

To further elucidate the driving mechanisms behind the taxonomic and functional profiles of oyster-associated microbiome along the Chinese coastline, we conducted a microcosm experiment using oysters to confirm the observed relationships between salinity and these microbial properties. We set three salinity levels (15, 25, and 30 ppt) to mimic the salinity conditions detected in this study and reflect the high salinity levels reported in a previous study [33]. Our results demonstrated that the bacterial composition varied significantly among the different salinity conditions (Fig. 5A; adonis *P* = 0.002), as indicated by PcoA. This suggests that salinity could affect oyster-associated microbiome. We quantified the abundance of key functional genes in oyster-associated microbiome related to C (*korA*, *frdA*, *aclB*, *abfA*, and *manB*), N (*napA*, *narG*, and *nirK*), and S (*dsrAB* and *suxY*) cycles. The abundance of all functional genes, including *korA*, *narG*, and *drsA*, increased initially as salinity rose from 15 ppt to 25 ppt, and then decreased as salinity increased from 25 ppt to 30 ppt (Fig. 5C). The C metabolism functions of oyster-associated microbiome exhibited a similar trend, with an initial increase (salinity from 15 ppt to 25 ppt, significantly increased by 21.7%), followed by a decrease (salinity from 25 ppt to 35 ppt, significantly decreased by 20.8%) (Fig. 5D; *P* < 0.05). These results indicate that salinity influences the functional profiles of oyster-associated microorganisms. Collectively, these findings highlight that salinity influences both the taxonomic and functional profiles of oyster-associated microbial communities and drives their spatial distribution patterns.

We examined the relationships between the taxonomic and functional profiles of oyster-associated microorganisms. We observed a strong relationship between the functional profiles and bacterial composition (Fig. 5F; M^2^ = 0.54 and *P* = 0.001), suggesting that bacterial composition significantly impacts functional profiles. *Vibrio* strains were identified as the most abundant microorganisms, accounting for 8.5%–85.7% of the total bacterial population (Fig. 5B), which was consistent with the results from metagenomic sequencing (Fig. 1F). The highest abundance of *Vibrio* was identified at 25 ppt (85.7%), significantly decreasing with increasing salinity from 25 ppt to 30 ppt. Furthermore, salinity also influences bacterial networks. Based on the degree of the bacterial networks, we observed a hump-like change in the stability and complexity of the bacterial networks as salinity increased from 15 ppt to 30 ppt. Specifically, stability and complexity first increased (salinity from 15 ppt to 25 ppt) and then decreased (salinity from 25 ppt to 30 ppt) (Fig. S7). These findings were consistent with the results of the survey (Fig. 2A and B; salinity: north: 18.3 ppt, south: 13.3 ppt). Additionally, *Vibrio* was well associated with other genera in microbial networks, especially in the 15 ppt and 25 ppt treatments (Table S4), suggesting *Vibrio* was the hub microorganism in these microbial networks, which is key to maintaining the stability and essential functions of ecological networks. We further examined the effects of varying salinity conditions (0–50 ppt) on the growth fitness of *Vibrio* strains, including *Vibrio alginolyticus*, *Vibrio neocaledonicus*, *Vibrio hyugaensis*, *Vibrio sp. O1*, and *Vibrio sp. O2*. We found that the fitness of *Vibrio* strains increased when salinity was below 20 ppt but started to decrease when salinity exceeded the threshold of 25 ppt (Fig. 5E). Consequently, the above results highlight the diverse growth adaptation strategies of oyster-associated microbiome under varying salinity conditions. An increase in suitable salinity will select for and enrich certain microorganisms, such as *Vibrio*, further affecting their metabolic pathways involved in the C, N, and S cycles. An increase in suitable salinity also enhances the complexity and stability of bacterial networks, which are critical for maintaining essential ecosystem services. However, a continuous increase will have the opposite effect. Notably, our results demonstrate that salinity-mediated microbial composition and microbial networks drive the spatial distribution patterns of functional profiles, such as C, N, and S cycles.

## Discussion

**Figure 6:**
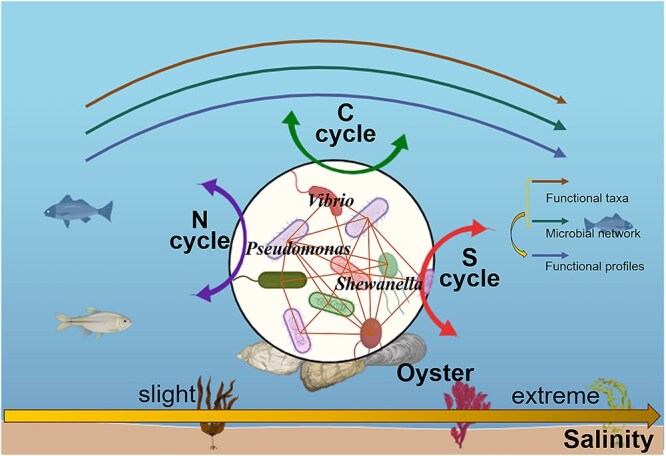
Conceptual paradigm showing the responses of the oyster-associated microbiome and its functional profiles under increasing salinity stress. The oyster-associated microbial communities and their functional traits alter with increasing salinity. With a slight increase in salinity, the complexity and stability of microbial ecological networks increase and certain functional microorganisms enrich, and then shaping the abundant carbon, nitrogen, and sulfur biogeochemical cycles. However, after exceeding the salinity threshold, excessive salinity will have the opposite effect on the above aspects, which is not conducive to these biogeochemical cycles.

Safeguarding coastal marine ecosystems is of paramount importance for facilitating material circulation and energy flow on Earth, and consequently contributing to global ecosystem health. Understanding the factors influencing the taxonomic and functional profiles of microbial communities is therefore essential [11, 12]. We performed the large-scale field survey to explore how environmental heterogeneity influence the biogeography and functional profiles of oyster-associated microbiome. This survey refined our understanding of environmental drivers, ensuring that conclusions are ecologically robust and practically relevant. Furthermore, we performed the microcosm experiment with oysters to further assess the effects of environmental conditions on the taxonomic and functional profiles of oyster-associated microorganisms. By combining a large-scale field survey with microcosm experiments, we developed a conceptual framework to summarize the ecological patterns of oyster-associated microbiome (Fig. 6). This contributes to a predictive understanding of host-associated microbial responses to the growing environmental stress in the Anthropocene. With increasing salinity stress, the oyster-associated microbiome underwent significant turnover. The microbial networks became more stable and complex with a slight increase in salinity, which was conducive to maintaining essential ecosystem services (e.g. C, N, and S biogeochemical cycles). A moderate increase in salinity further selected and enriched certain functionally relevant microorganisms (e.g. *Vibrio*) and affected the metabolic pathways involved in the C, N, and S cycles. However, after exceeding the salinity threshold, excessive salinity had the opposite effect. Importantly, salinity-driven microbial networks and composition, especially functional taxa, played predominant roles in shaping the functional profiles of oyster-associated microorganisms. Our results revealed that salinity mediates the spatial distribution patterns of the taxonomic and functional profiles of oyster-associated microorganisms along the Chinese coastline.

Oysters are widely distributed and cultured along the 18 000-km coastline of China [4]. Recently, host-associated microbiome has been increasingly recognized as essential contributors to ecosystem services and functioning, including the remineralization of organic materials and the transformation of C, N, and S into inorganic forms [8, 9]. In this study, we identified abundant functional microorganisms and their biological processes in oyster-associated microorganisms, suggesting that oyster-associated microorganisms play an important role in maintaining the health of coastal marine ecosystems [8]. In this study, we found that oyster species had the small effect on oyster-associated microorganisms (Fig. S10). However, it is important to note that oyster species may still influence the taxonomic and functional profiles of oyster-associated microbiome, which warrants further investigation.

The taxonomic and functional profiles of the oyster-associated microbial communities exhibited spatial heterogeneity from northern to southern regions. This was consistent with the results of previous studies [14, 22, 23]. Interestingly, there was a distinct variation in salinity between the northern and southern maricultural regions (Fig. S1). The effects of salinity on microbial community structure and function have been widely reported. For example, salinity stress has been found to significantly alter gut and stomach microbial communities in fish and mud crabs [19–21]. Consistent with these studies, our study identified salinity, the most significant variable in coastal marine areas [40], as the primary regulator of the spatial distribution of taxonomic and functional oyster-associated microbiome. Furthermore, microbial networks can provide insights into the complex and stable microbial interactions that are critical for maintaining essential ecosystem services [41]. Numerous studies have revealed that increased salinity stress diminishes the complexity and stability of microbial co-occurrence networks in various habitats, such as estuaries, soils, and wastewater [13, 42, 43], because only microorganisms adapted to these specific conditions can thrive under extreme salinity conditions [13]. However, we observed that higher salinity (northern: 18.3 ppt) resulted in relatively stable and decentralized microbial networks compared to those of lower salinity (southern: 13.3 ppt) environments (Fig. 2). This finding is related to specific environmental stresses associated with salinity conditions [16]. Fluctuations in appropriate salinity can reduce the number of species or types of microorganisms, while the remaining microorganisms may promote the establishment of a more interconnected community by sharing resources and forming cooperative relationships to withstand environmental stress [44, 45]. Similar results have been reported for groundwater and sediments [18, 46]. The microbial network complexity in our study displayed a hump-like change with increasing salinity, which may contribute differently to ecosystem stability and functions, such as organic matter transformation [46, 47]. However, further studies with a larger sample size are still needed to validate these findings.

When salinity levels increase, numerous microorganisms become inactive owing to elevated extracellular osmotic pressure [48] and the production of reactive oxygen species [49]. Therefore, we observed significantly lower oyster-associated microbial diversity in the northern regions (salinity: 18.3 ppt) than in the southern (salinity: 13.3 ppt) regions (Figs. 1B and C, S8; *P* < 0.05). However, other microorganisms may exhibit a relatively high tolerance to suitably elevated salinity and flourish [50]. Certain members of *Vibrio*, *Pseudoalteromonas*, *Pseudomonas*, and *Shewanella* have been identified as salt-tolerant microorganisms [51, 52] and were extensively detected in this study (Fig. 4C). The presence of related genes in these MAGs confirmed that they are key functional microorganisms (Fig. 4C), and their metabolic mutualism among different functional members plays a significant role in the coupled C, N, S, and iron biogeochemical cycles. These functional MAGs were more abundant in the northern regions than in the southern regions (Figs. 4C and S4; *P* < 0.05), which may have led to the enrichment of functional profiles in the northern regions. However, associated functional microorganisms, such as *Vibrio*, need to be isolated for further study to explore their specific functions; this planed for our future research. Additionally, faced with the challenge of salinity stress, microorganisms can employ varied adaptation strategies, including “salt-in” and “salt-out,” to aid targeted survival efforts [53]. Microorganisms must increase energy expenditure and utilize a broader range of C sources to synthesize and/or uptake compatible solutes from the environment to withstand salinity stress. For example, certain members of *Vibrio* can synthesize polysaccharides and proteins, utilize quorum-sensing properties to endure salinity stress, and simultaneously affect the nitrification process [54]. A similar study reported that the intestinal microbiota of aquatic organisms can synthesize associated metabolites, such as cellulose or chitin, after acute salt stress [19]. Previous studies have demonstrated that moderate salinity promotes nitrification mediated by ammonia-oxidizing bacteria [55], N_2_O emissions [56], sulfate reduction [18], and CH_4_ release [57]. However, extreme salinity decreases the rates of nitrification, carbohydrate metabolism, and methanogenesis [18, 58, 59]. Therefore, varied salinity conditions can affect the energy costs and metabolic pathways of oyster-associated microorganisms [60], potentially promoting or inhibiting their specific functional processes [61]. However, the mechanisms by which salinity affects the functional processes of oyster-associated microorganisms involved in the C, N, and S cycles require further exploration.

Salinity is one of the most crucial parameters reflecting the prevailing conditions of marginal marine environments [40] and is particularly sensitive to climate change. Recent studies have predicted that salinity in certain ocean regions will increase owing to evaporation [62, 63]. Considering the impacts of climate change, particularly changes in salinity, oyster-associated microorganisms and their functional profiles will become increasingly vulnerable, posing significant risks to seafood safety (e.g. quality and yield) and the health of global ecosystems (e.g. C, N, and S cycles). Therefore, it is crucial to pay more attention to the effects of climate change on both seafood safety and coastal ecosystem health.

## Supplementary Material

supplementary_ycaf080

## Data Availability

The sequenced data was deposited in NCBI under the accession number PRJNA1056564.
