# Supplementary material for: Salinity drives the biogeography and functional profiles of the oyster-associated microbiome along the Chinese coastline
Source: ISME Commun. 2025 May 9;5(1):ycaf080. doi: 10.1093/ismeco/ycaf080 (PMC12445697; doi:10.1093/ismeco/ycaf080)
Supplement: supplementary_ycaf080 [file supplementary_ycaf080.pdf]

Salinity drives the biogeography and functional profiles of the oyster-associated microbiome along the Chinese coastline

Liusheng Lei [1], Xin Li [1], Linhao Chen [1], Xiaolong Wang [1], Qingbin Yuan [1], Zeyou Chen [2], Daqing Mao [3], Yi Luo [1*], and Huai Lin [1 *]

Affiliations and addresses:

[1] *State Key Laboratory of Water Pollution Control and Green Resource Recycling, School of the Environment, Nanjing University, Nanjing 210093, China*

[2] *Ministry of Education Key Laboratory of Pollution Processes and Environmental Criteria, College of Environmental Science and Engineering, Nankai University, Tianjin 300350, China*

[3] *School of Medicine, Nankai University, Tianjin 300310, China*

***Corresponding author:**

Huai Lin (linhuai@nju.edu.cn); Yi Luo (luoy@nju.edu.cn)

Figure and table of contents

Section I. 16S rRNA gene sequencing

Section II. Quantify of key functional genes related to C, N, and S cycles

Section III. Biolog EcoPlate inoculation and analyses

Section IV. *Vibrio* strains isolated from oysters

Section V. Growth curve measurement of isolated *Vibrio* strains

Figure S1. The relative abundance of domnant bacterial and archaeal genus between southern and northern regions. Significant differences: * $P < 0.05$ and *** $P < 0.001$.

Figure S2. Physicochemical parameters of sampling sites between southern and northern regions, incuding salinity, temperature, pH, TN, TP, COD, NH_4^+-N, and Fe^{3+}. Significant differences: * $P < 0.05$.

Figure S3. The abundance of all functional pathways of oyster-associated microbes between southern and northern regions.

Figure S4. Abundance of good-quality metagenomic assembled genomes and their carried the carbon, nitrogen, and sulfur cycling genes.

Figure S5. The effects of different factors on functional taxa of oyster-associated microbes according to mantel test. Different factors include incuding salinity, temperature, pH, TN, TP, COD, NH_4^+-N, and Fe^{3+}. Significant differences: * $P < 0.05$, ** $P < 0.01$, and *** $P < 0.01$.

Figure S6. Heatmap of the C, N, S cycle of functional genes carried by functional microorganisms (MAGs) between northern and southern regions. Genes belonging to the same function are depicted using the consistent color. C cycle includes C fixation, C degradation, and CH_4 metabolism. C: carbon; CH_4: methane; N: nitrogen; S: sulfur.

Figure S7. The co-occurrence networks of oyster-associated bacterial communities in different salinity conditions according to Pearson's analysis. Different salinity conditions: 15, 25, and 30 ppt. The nodes are colored according to modularity and node size is proportional to node degree. A connection served as a significant correlation (R > |0.9|, P < 0.05).

Figure S8. Bacterial and archaeal diversity between northern and southern regions. Significant differences: *** P < 0.01.

Figure S9. The abundance of oyster-associated dominant bacterial (a) and archaeal (b) phyla along Chinese coastline.

Figure S10 Contribution of various factors such as salinity, temperature, Fe^{3+}, and oyster species to microbial composition using redundancy analysis.

Figure S11 The correlation analysis between geography and environmental factors such as salinity, temperature, pH, COD, TP, Fe^{3+}, NH_4^+-N, and TN based on the mantel tests. Color gradient and circle size denote Pearson's correlation coefficients. The color of the line represents

the significance of the differences (P values). The size of the line represents correlation coefficients (mantel's r). Asterisks in the circle denote for different significance levels at $*P < 0.05$, $**P < 0.01$, and $***P < 0.001$.

Figure S12 The correlation analysis between geography and environmental factors such as salinity, temperature, pH, COD, TP, Fe^{3+}, NH_4^+-N, and TN based on the mantel tests. Color gradient and circle size denote Pearson's correlation coefficients. The color of the line represents the significance of the differences (P values). The size of the line represents correlation coefficients (mantel's r). Asterisks in the circle denote for different significance levels at $*P < 0.05$, $**P < 0.01$, and $***P < 0.001$.

Table S1 The primer sequences and annealing temperature for each target gene used in this study.

Table S2 Details of oyster samples from different sites among coastline of China.

Table S3 Detailed information on the physicochemical properties of the sampling sites.

Table S4 The degree of *Vibrio* in microbial networks.

Section I. 16S rRNA gene sequencing

Oyster samples were ground using tissue grinder and extracted the total genomic DNA using E.Z.N.A. Stool DNA Kit (Omega Biotek, CA) according to manufacturer's instructions. DNA was examined using 1.0% (v/v) agarose gel electrophoresis and quantifed using Nanodrop 2000 (Thermo Scientific, Waltham, MA). For 16S rRNA gene libraries, the V3-V4 region was amplifed using the universal primers 5'-ACTCCTACGGGAGGCAGCA-3' and 5'-GGACTACHVGGGTWTCTAAT-3'. The 16S rRNA gene sequencing was performed on the Illumina NorvaSeq 6000 platform with 150 bp paired-end technology. The sequenced data was processed for quality control using Trimmomatic v0.33 and cutadapt 1.9.1. After removing barcodes and primers, low-quality reads were fltered, and the non-chimeric reads were identified using dada2. After quality trimming, the taxonomy of each sequence was analyzed by RDP Classifer against the SILVA small subunit rRNA database (version 138).

Section II. Quantify of key functional genes related to C, N, and S cycles

The key genes related to C (*naglu, manB, abfA, amyA, accA, aclB, frdA*, and *korA*), N (*glnA, napA, narG, nirK*), and S (*dsrAB* and *soxY*) cycles were selected and quantified using qPCR. The qPCR protocol was as follows: initial denaturation for 10 min at 95 °C, followed by 40 cycles at

95 °C for 10 s, annealing at the appropriate temperature for 30 s, and extension at 72 °C for 30 s (Table S1). The amplification efficiencies calculated ranged between 90% and 110%.

Section III. Biolog EcoPlate inoculation and analyses

Two gram of oyster samples were ground using tissue grinder and suspended into 18 mL of sterilized NaCl solution (0.85%, w/v). These suspensions were shaken for 20 min at 20 °C. Ten-fold serial dilutions were prepared and the 10^{-3} dilution was used to inoculate the plates. 150μL of the diluted suspension was added to 96 plate wells. Subsequently, the plates were cultivated at 25 °C in darkness for 72 h. The absorbance of plates was detected and recorded at 590 nm and 750 nm per 24 h. Optical density value from each well was adjusted by subtracting the absorbance of the control value. The 24-h incubation results were used to assess the carbon source utilizaton by cacluating the average well-color developments

Section IV. *Vibrio* strains isolated from oysters

All oyster samples (~5 g) and 5 mL sterile phosphate-buffered saline (PPS) were ground into suspensions using tissue grinder (70 HZ and 60 s). The suspensions were then transferred into 50-mL sterile water tubes and maintained at 25 °C and 4000 rpm for 5 min. The supernates were serially

diluted in 9 mL sterile PPS, and then 100 μL resultant suspensions were plated on thiosulfate citrate bile salt sucrose (TCBS) agar media isolate the *Vibrio* strains. All samples were prepared in triplicate. All TCBS plated were incubated at 37°C for 1-2 days. Colonies were selected according to different colors and colony morphology, and incubated in liquid LB with at 37 °C overnight. 16S rRNA gene were detected using universal primers (27F and 1492R) for identification of the selective strains.

Section V. Growth curve measurement of isolated *Vibrio* strains

Growth curves measurements of *Vibrio* strains were conducted in the presence of different levels of salinity. The NaCl was added to the bacterial solution that contained 10^6 CFU/mL of *Vibrio* strains in 96-well microtitet plates, to achieve final salinity levels of 0, 5, 10, 15, 20, 25, 30, 35, 40, 45, and 50 ppt. These plates were incubated at 37°C for 24 h and measured OD600 value at each 30 min to obtain the growth curves. Each condition was prepared in triplicate. Growth curve parameters, including growth rates, were calculated using the R package "Growthcurve" (https://cran.r-project.org/web/packages/growthcurver/index.html).

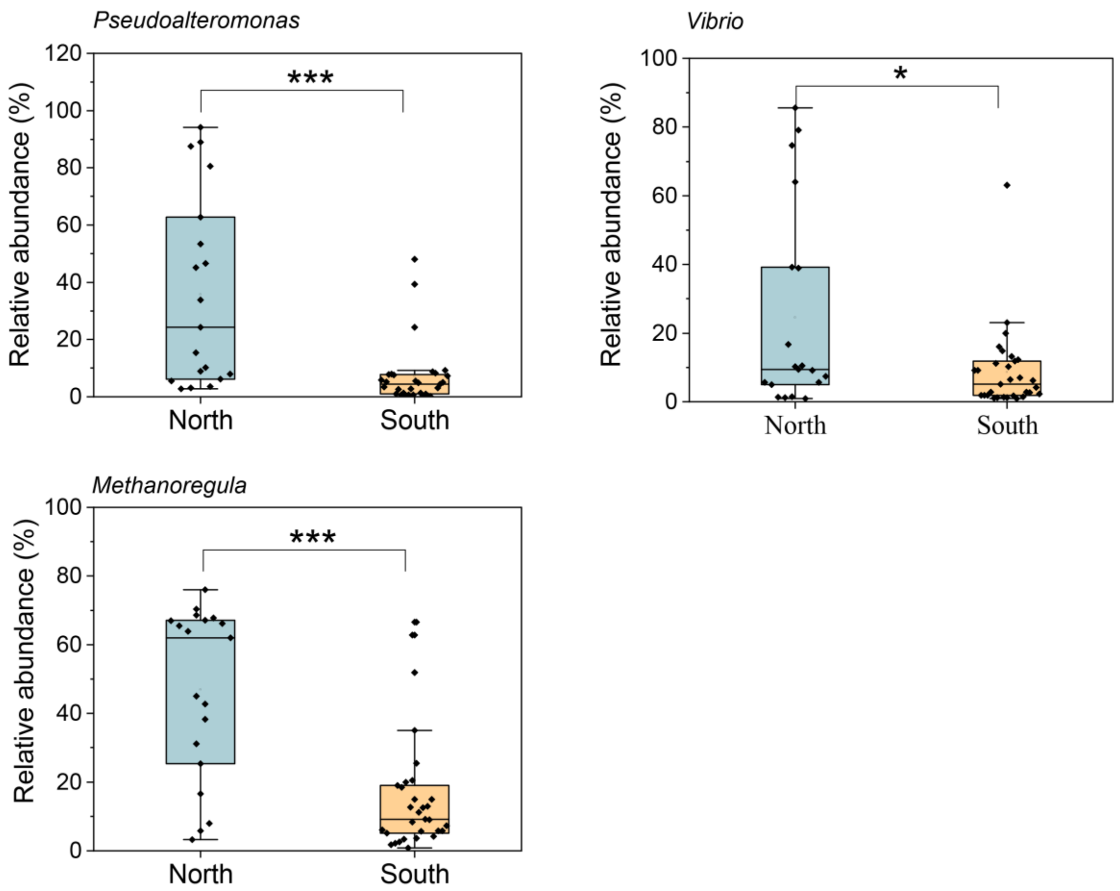

Figure S1. The relative abundance of domnant bacterial and archaeal genus between southern and northern regions. Significant differences: * $P < 0.05$ and *** $P < 0.001$.

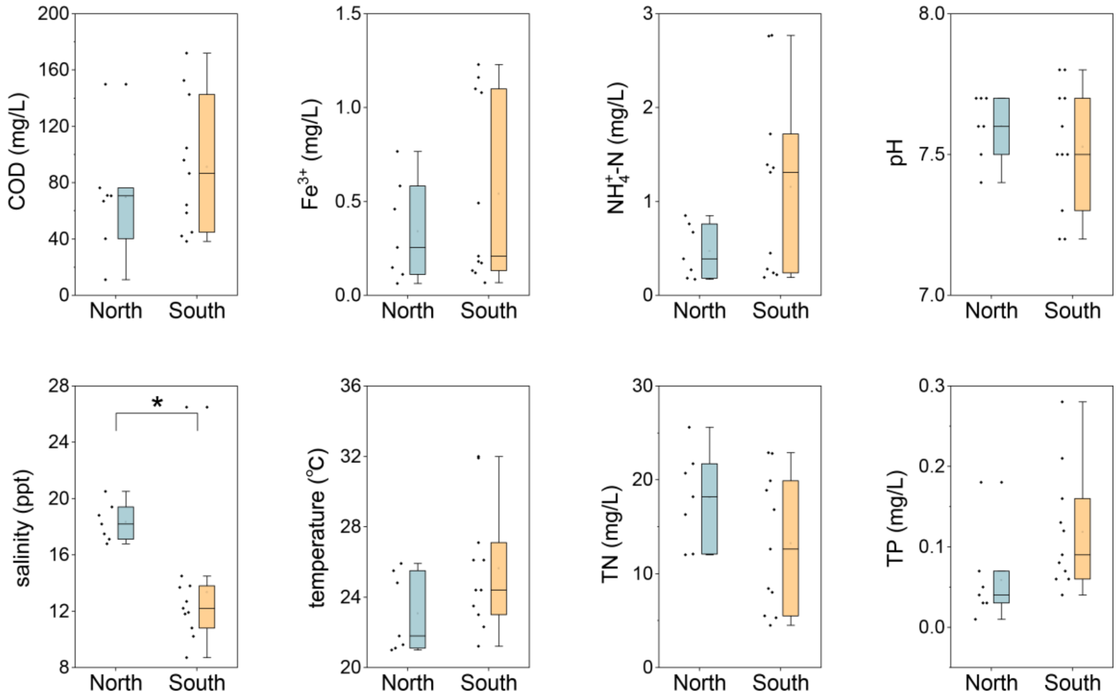

Figure S2. Physicochemical parameters of sampling sites between southern and northern regions, incuding salinity, temperature, pH, TN, TP, COD, NH_4^+-N, and Fe^{3+}. Significant differences: * P < 0.05.

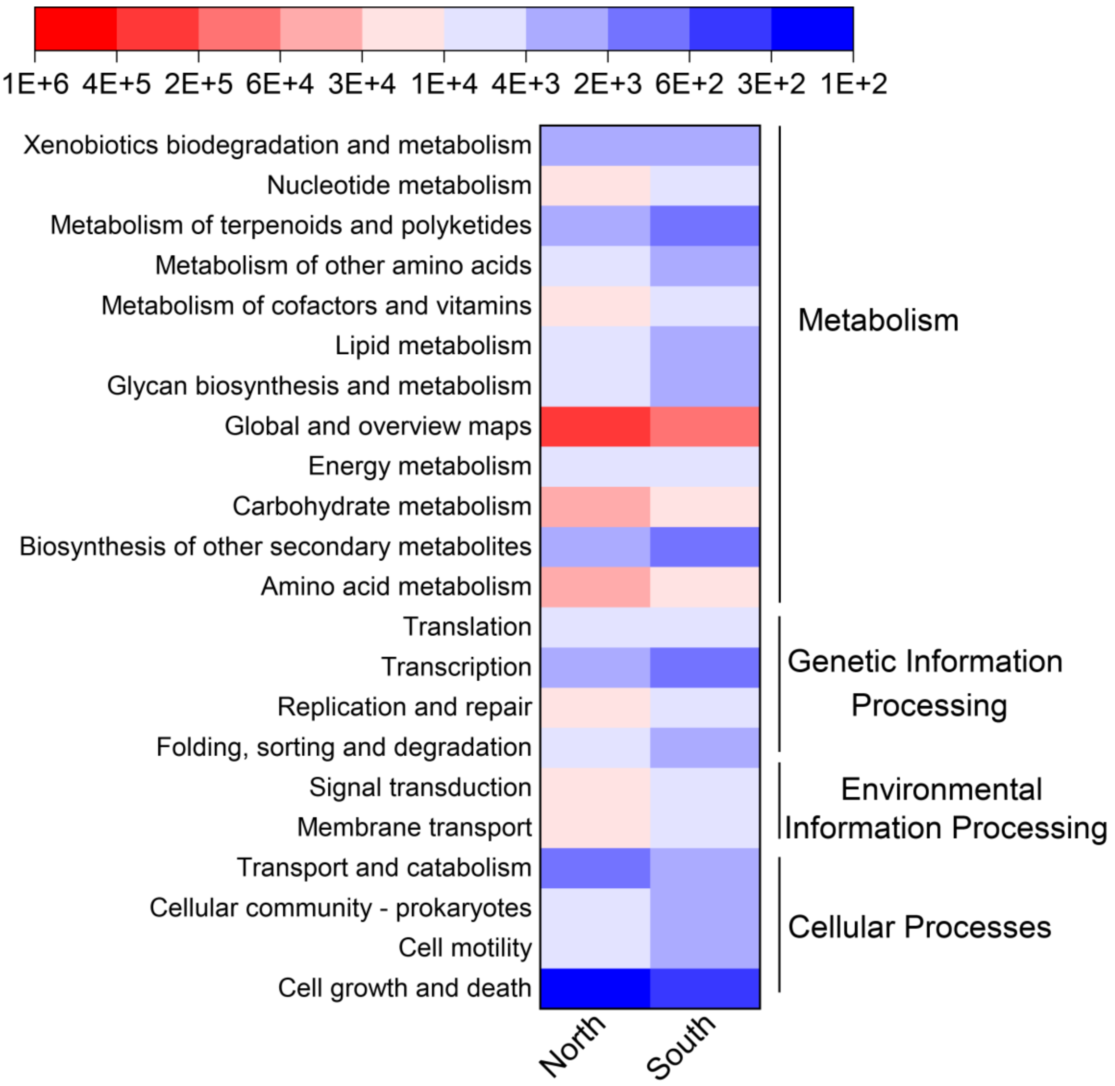

Figure S3. The abundance of all functional pathways of oyster-associated microbes between southern and northern regions.

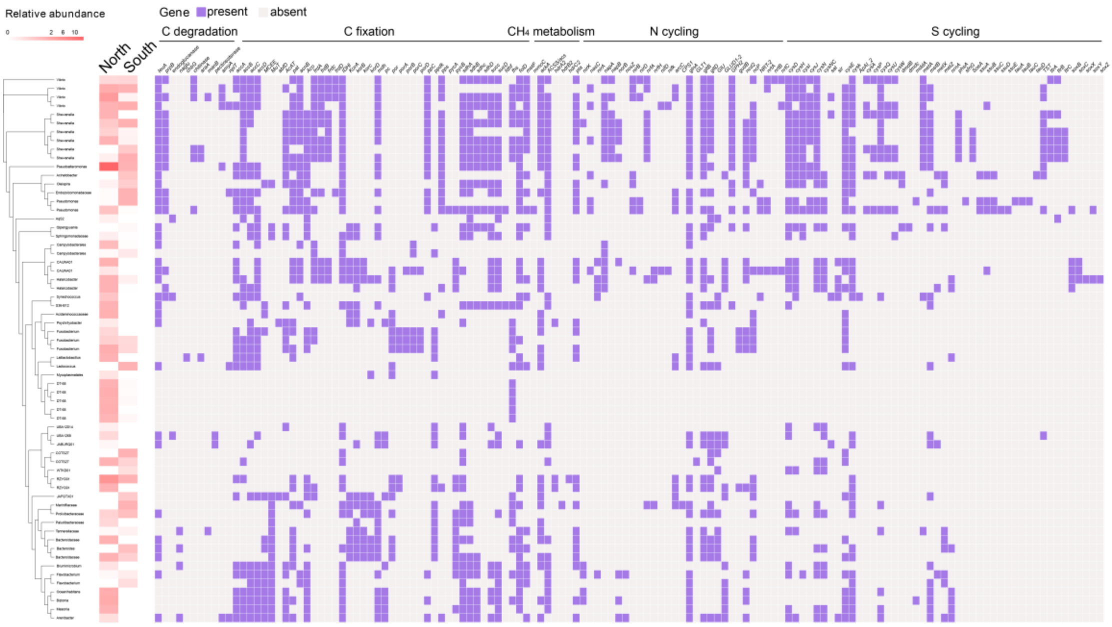

Figure S4. Abundance of good-quality metagenomic assembled genomes (MAGs) and their carried the carbon, nitrogen, and sulfur cycling genes.

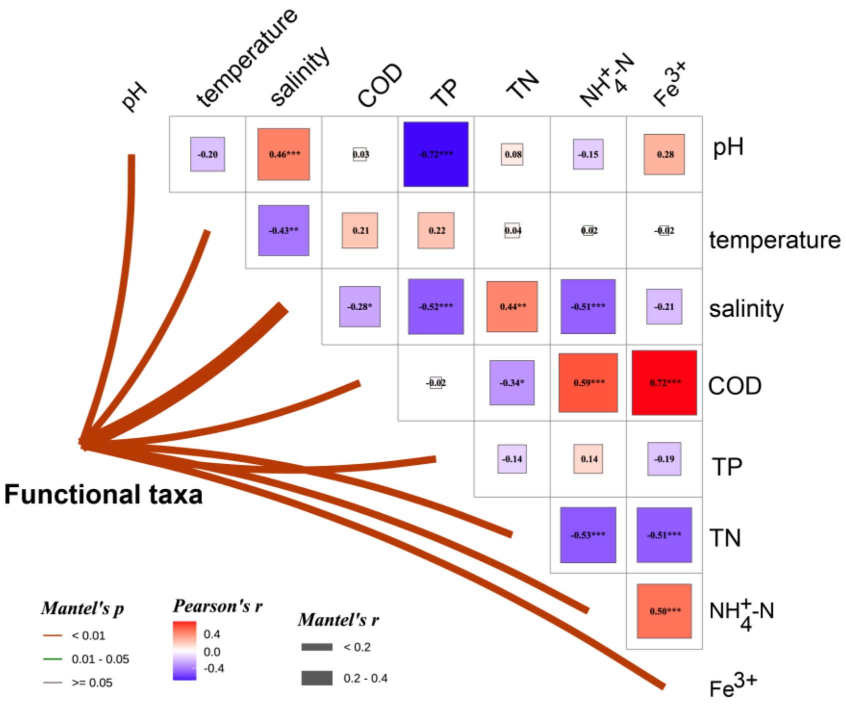

Figure S5. The effects of different factors on functional taxa of oyster-associated microbes according to mantel test. Different factors include incuding salinity, temperature, pH, TN, TP, COD, NH_4^+-N, and Fe^{3+}. Significant differences: * $P < 0.05$, ** $P < 0.01$, and *** $P < 0.01$.

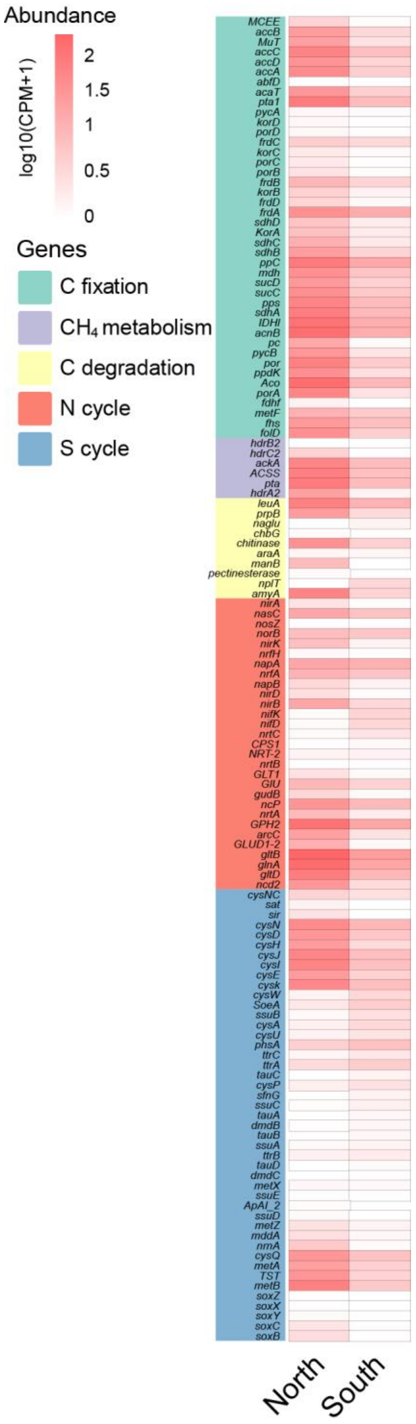

Figure S6. Heatmap of the C, N, S cycle of functional genes carried by functional microorganisms (MAGs) between northern and southern regions. Genes belonging to the same function are depicted using the consistent color. C cycle includes C fixation, C degradation, and CH_4 metabolism. C: carbon; CH_4: methane; N: nitrogen; S: sulfur.

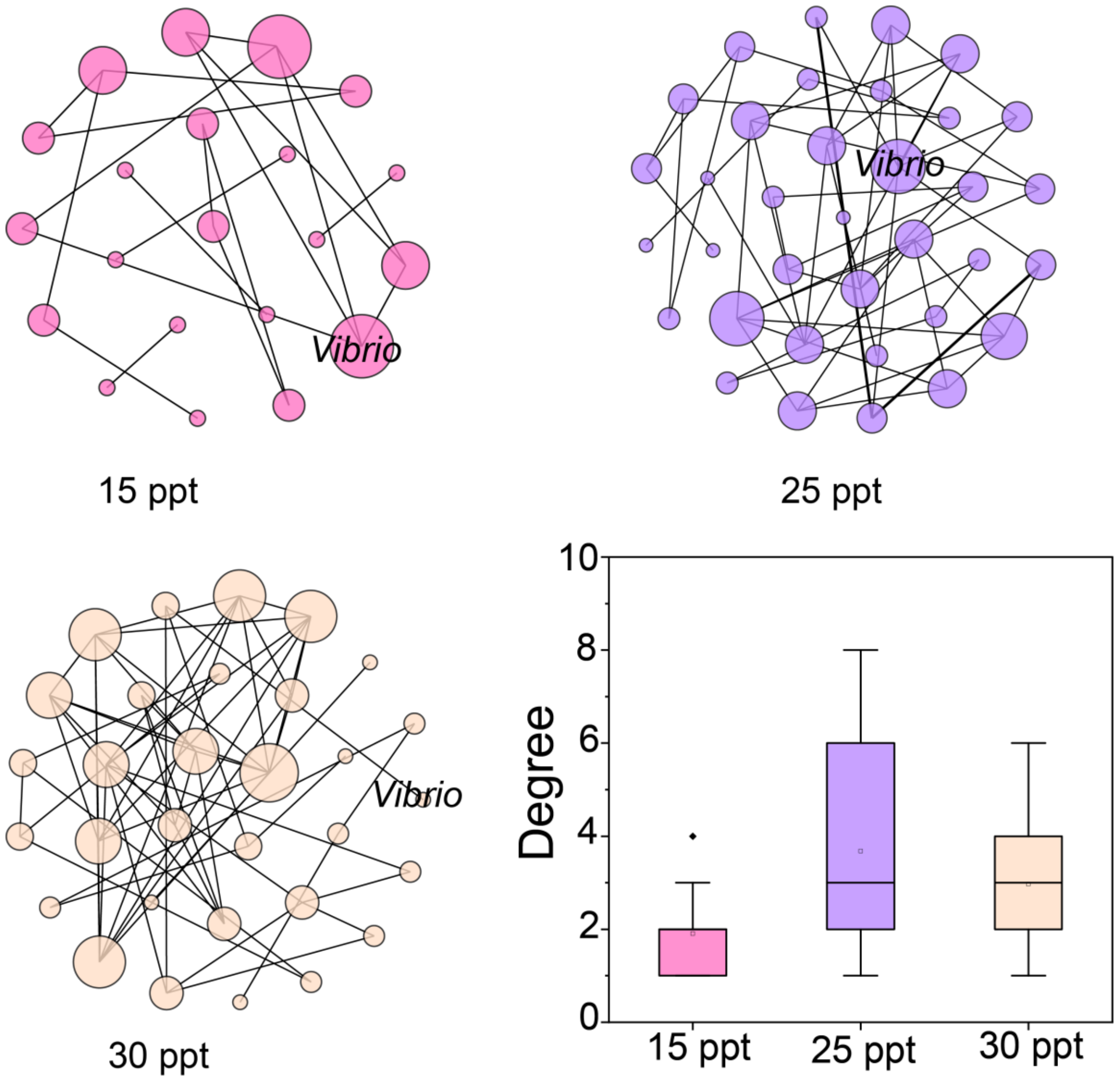

Figure S7. The co-occurrence networks of oyster-associated bacterial communities in different salinity conditions according to Pearson's analysis. Different salinity conditions: 15, 25, and 30 ppt. The nodes are colored according to modularity and node size is proportional to node degree. A connection served as a significant correlation (R > |0.9|, *P* < 0.05).

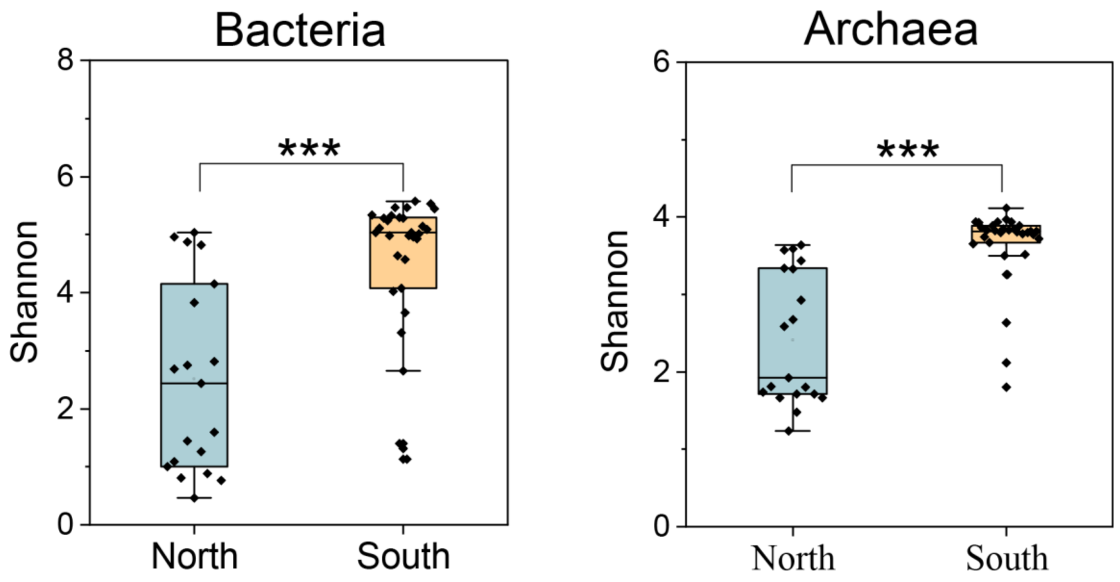

Figure S8. Bacterial and archaeal diversity between northern and southern regions. Significant differences: *** $P < 0.01$.

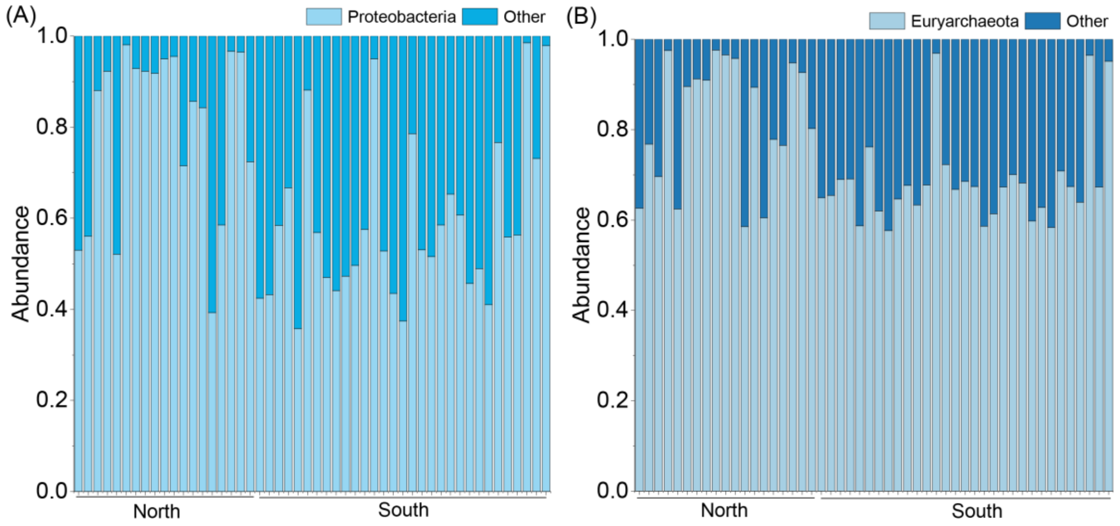

Figure S9. The abundance of oyster-associated dominant bacterial (a) and archaeal (b) phyla along Chinese coastline.

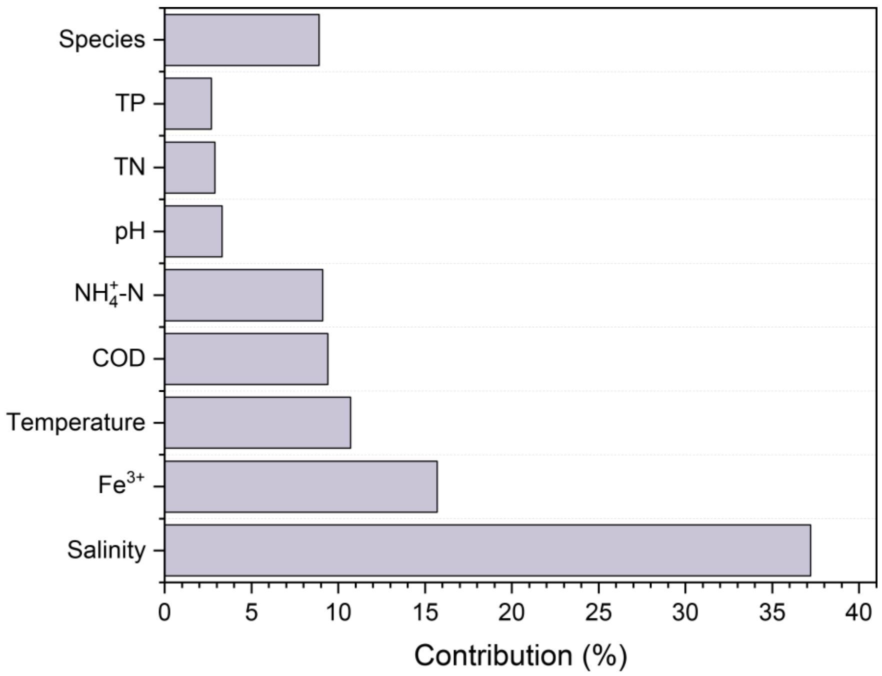

Figure S10 Contribution of various factors such as salinity, temperature, Fe^{3+}, and oyster species to microbial composition using redundancy analysis.

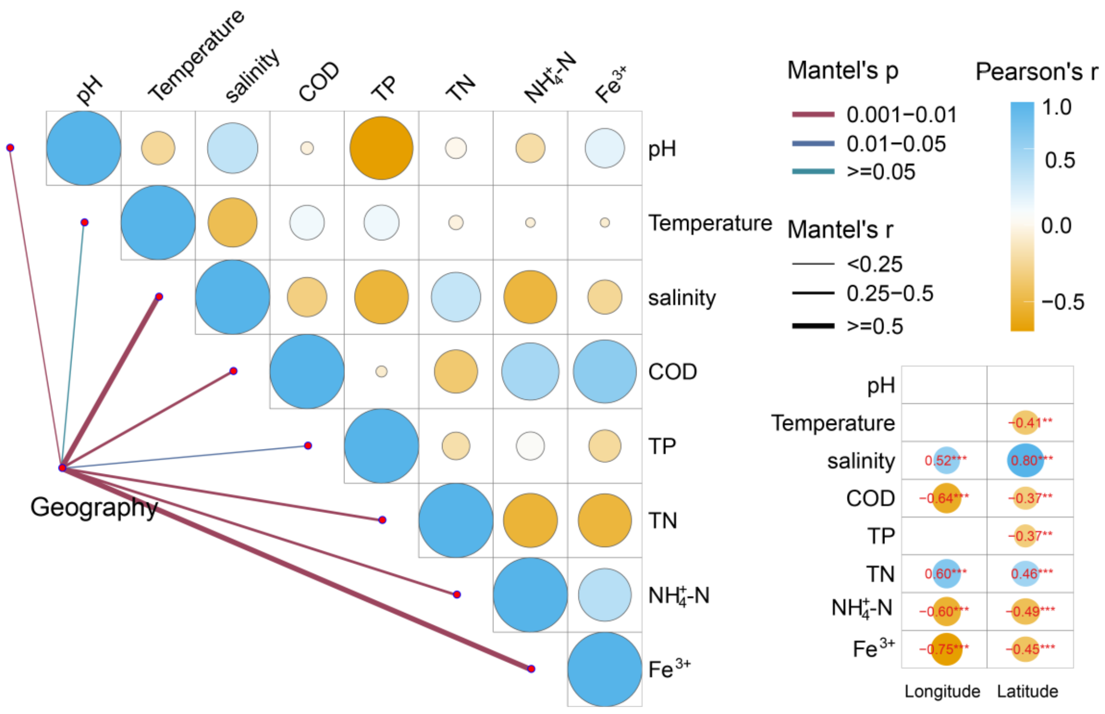

Figure S11 The correlation analysis between geography and environmental factors such as salinity, temperature, pH, COD, TP, Fe^{3+}, NH_4^+-N, and TN based on the mantel tests. Color gradient and circle size denote Pearson's correlation coefficients. The color of the line represents the significance of the differences (P values). The size of the line represents correlation coefficients (mantel's r). Asterisks in the circle denote for different significance levels at **P* < 0.05, ***P* < 0.01, and ****P* < 0.001.

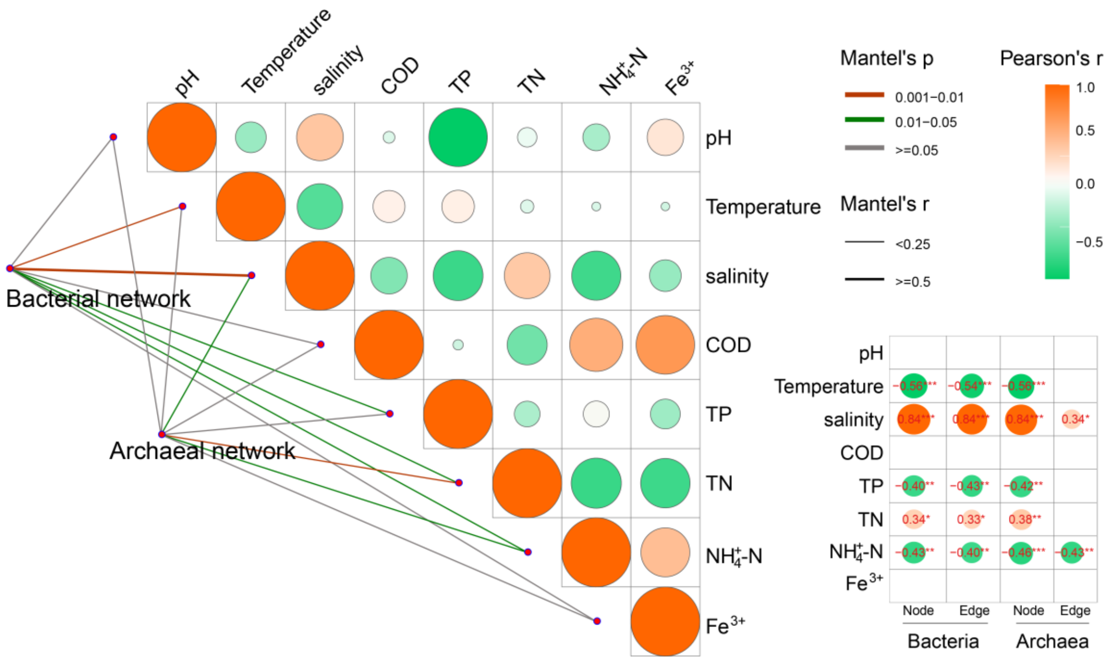

Figure S12 The correlation analysis between geography and environmental factors such as salinity, temperature, pH, COD, TP, Fe^{3+}, NH_4^+-N, and TN based on the mantel tests. Color gradient and circle size denote Pearson's correlation coefficients. The color of the line represents the significance of the differences (P values). The size of the line represents correlation coefficients (mantel's r). Asterisks in the circle denote for different significance levels at *P < 0.05, **P < 0.01, and ***P < 0.001.

Table S1 The primer sequences and annealing temperature for each target gene used in this study.

Gene name	Primer	Annealing temperature (°C)
narG	F: CTCGAYCTGGTGGTYGA<br>R: TTYTCGTACCAGGTSGC	54
napA	F: AAYATGGCVGARATGCACCC<br>R: GRTTRAARCCCATSGTCCA	58
nirK	F: ATCATGGTSCTGCCGCG<br>R: GCCTCGATCAGRTTGTGGTT	58
glnA	F: AATCATGTTCGACGGCTCT<br>R: GGCAACTTTCCCTTTCTCA	60
dsrA	F:CGCRACGGCAASAAGGTSMSSGT<br>R: CAKRTGCAKSGCRTGGCAGAA	58
dsrB	F: CAACATCGTYCAYACCCAGGG<br>R: GTGTAGCAGTTACCGCA	58
soxY	F: ATCGATGACAACCCCGTGCC<br>R: AGCTGGTCCATCTGCATGCCG	58
naglu	F: TVAAYTGGTAYCTGAAATAY<br>R: CCRTGYAGVGCCATCCAGTC	58
manB	F: ATGCGCGGBGTCAACCA<br>R: TCGTTGSCGATGTTGABGA	58
abfA	F: CGSTAYCCSGGCGGCAAYTT<br>R: TGCCASGGNCCGTCCATYTC	58
amyA	F: YGGTTTTCGTCTTGACGCSG<br>R: MGGCTGMGTRTCATGRTTK	58
accA	F: GAAGGCTAYCGCAARGC<br>R: CCTTCMGGSGARATMA	58
aclB	F: TGGACMATGGTDGCYGGKGGT<br>R: ATAGTTKGGSCCACCTCTTC	58
frdA	F: MTGCTGCACACSCTGTW<br>R: CCGGTSGGGTGRWACTG	58
korA	F: GCCGGCTACCCCATCACCCC<br>R: ATGATGGGATGGTCGCCATG	58

Table S2 Details of oyster samples from different sites among coastline of China.

Sites	Longitude	Latitude	Province	Sea area	Oyster species	Number
S1	122.97	39.56	Liaoning	Yeallow Sea	*Crassostrea gigas*	3
S2	122.68	39.28	Liaoning	Yeallow Sea	*Crassostrea gigas*	3
S3	120.35	37.75	Shandong	Bohai Sea	*Crassostrea gigas*	3
S4	121.41	37.58	Shandong	Yeallow Sea	*Crassostrea gigas*	3
S5	121.40	36.76	Shandong	Yeallow Sea	*Crassostrea gigas*	3
S6	121.53	36.68	Shandong	Yeallow Sea	*Crassostrea gigas*	3
S7	120.06	35.85	Shandong	Yeallow Sea	*Crassostrea gigas*	3
S8	121.79	29.66	Zhejiang	East China Sea	*Crassostrea sikamea*	3
S9	121.57	29.05	Zhejiang	East China Sea	*Crassostrea sikamea*	3
S10	121.54	29.03	Zhejiang	East China Sea	*Crassostrea sikamea*	3
S11	121.07	28.28	Zhejiang	East China Sea	*Crossostrea angulate*	3
S12	121.13	28.25	Zhejiang	East China Sea	*Crossostrea angulate*	3
S13	120.53	27.25	Zhejiang	East China Sea	*Crassotrea sikamea*	3
S14	117.25	23.64	Fujian	South China Sea	*Crassostrea angulate*	3
S15	114.87	22.74	Guandong	South China Sea	*Crassostrea hongkongensis*	3
S16	110.42	21.36	Guandong	South China Sea	*Crassostrea hongkongensis*	3
S17	108.60	21.84	Guanxi	South China Sea	*Crassostrea hongkongensis*	3
S18	108.69	21.69	Guanxi	South China Sea	*Crassostrea hongkongensis*	3

Table S3 Detailed information on the physicochemical properties of the sampling sites.

Sites	pH	Temperature (℃)	Salinity (ppt)	COD (mg/L)	TP (mg/L)	TN (mg/L)	NH_4^+-N (mg/L)	Fe^{3+} (mg/L)
S1	7.4	24.8	16.8	150.0	0.1	20.7	0.8	0.8
S2	7.6	21.8	20.5	70.9	0.1	21.7	0.3	0.1
S3	7.6	21.1	17.5	66.8	0.2	18.2	0.2	0.1
S4	7.5	21.3	17.1	11.1	0.0	16.3	0.9	0.3
S5	7.7	21.0	18.2	40.2	0.0	12.1	0.7	0.6
S6	7.7	25.5	19.4	70.6	0.0	12.0	0.2	0.5
S7	7.7	25.9	18.8	76.4	0.0	25.6	0.4	0.1
S8	7.7	31.9	12.7	58.4	0.1	22.8	0.5	0.5
S9	7.5	32.0	11.8	104.5	0.2	12.6	1.3	0.2
S10	7.3	27.1	11.9	38.3	0.1	8.0	0.3	0.1
S11	7.5	23.0	12.2	64.2	0.1	22.9	1.7	0.2
S12	7.6	23.5	13.8	42.1	0.1	8.4	1.4	0.2
S13	7.5	21.2	14.5	86.6	0.1	19.9	0.2	0.1
S14	7.2	26.1	10.8	44.7	0.3	18.9	0.2	0.1
S15	7.8	26.1	26.5	95.9	0.1	16.8	0.2	1.2
S16	7.2	22.3	10.2	152.5	0.2	5.5	2.8	1.1
S17	7.7	24.4	8.7	142.5	0.0	4.5	1.4	1.1
S18	7.8	24.4	13.7	172.1	0.1	5.3	2.8	1.2

Table S4 The degree of *Vibrio* in microbial networks.

Treatment	rank	degree
15 ppt	1	4
25 ppt	2	5
30 ppt	27	1
